# The "One Portal" Principle in Nursing

**Published:** 1909-07-24

**Authors:** 


### Nursing Administration.

### THE "ONE PORTAL" PRINCIPLE IN NURSING.

The one thing to be desired in the present stage of the movement in favour of registration is that free discussion on the subject should be promoted from all points of view. The very complex problems to be solved will not melt away under vehement denials that they exist, nor will the far seeing objections of women who have spent their lives in training nurses be overcome by tenacious declarations that these objections are based on ignorance and cowardice. All that kind of talk needs to be got rid of. It has to be clearly brought into the open that the registration of nurses in this country is exposed to many and grave drawbacks, and if it is to be brought about at all attention must be concentrated on the steps necessary to provide against passing from an admittedly imperfect state of affairs in the present, to one still more unsatisfactory because cast in an iron mould by legislation in the future.

Nothing has done more to make propositions for legislation acceptable than the eminently sane and practical discussions which have taken place on the subject over the border. The Association of which Lord Inverclyde is President has succeeded in uniting a very strong body of nursing opinion in Scotland in favour of a measure for regulating the nursing profession, and it is the desire of many who have hitherto been altogether discouraged about the proposed Bills to see an equally representative association formed from among hospitals south of the Tweed. At a conference recently held in London the principle of a single portal system of admission to the register for the whole of the United Kingdom was assented to, and there can be no doubt that if unity can be attained without the necessity for rigid uniformity, there would be a considerable gain for the nurses and the public. But it is necessary to scrutinise very closely the exact meaning of the expression which is being so freely used this week, "the single portal."

A central registering body is not incompatible with the principle of administrative decentralisation in regard to the examinations. The inspection and supervision of examinations by this central body need not carry with it an inflexible system of uniformity such as is practicable in the case of midwives trained for a few months only, and for one special branch of work. If the one portal system is to be taken as meaning that one set of papers is to be the passport for the nursing profession under whatever system, and in whatever locality the candidate may have been trained, we unhesitatingly maintain that the interests of the profession must inevitably suffer, and the standard of nursing deteriorate under its effects. Who can compare the experience afforded in the large clinical hospitals which can fully occupy their pupils during a four years' course, to the training given in small provincial hospitals which are nevertheless capable, especially when encouraged by competition and inspection, of turning out nurses thoroughly competent to undertake the ordinary duties of the sick-room? It is neither natural nor desirable in the interests of nurses trained under such widely different conditions as regards opportunity, to submit them to precisely similar examinations, as the passport to their profession. If the examinations are brought down to the level of such mediocre attainments as may be rightly expected from the one class of candidate, they cannot fail to discourage the other, who will rebel against the extra year's training, and other requirements tending to make British nurses the best trained members of the profession in the world. Who would not see the absurdity of setting the same examinations before the pass candidate of the schools and the honour students in classics, mathematics, medicine or science? The varying examinations all admit to the degree of the university, but no purpose would be served by pretending that the standard of knowledge was the same in all cases. It is undoubtedly desirable that nurses should have some practice in obstetric cases. But is it practicable to insist as is done in New York that each candidate shall have been afforded the opportunity of nursing a given number of abdominal cases as an essential preliminary to examination? Suppose that as a natural consequence of improved methods of midwifery the abdominal sections become rarer as time goes on? This is well within the bounds of hope, but are small training schools to be disqualified because women improve in health? Yet if obstetric work is excluded altogether from the examinations, it will be hard to keep those wards up to their present pitch of excellence. We mention this merely as an example of the many minor difficulties which crop up whenever the syllabus of one uniform examination is brought under discussion. We want before everything to keep the present splendid work done in the major training schools from suffering injury. We want, and this is scarcely less important, to improve the smaller training schools, and utilise all the material existing under a bewildering variety of organisation for the training of nurses. The two are incompatible under any uniform system of examination, and hence we deprecate the attempt to make this impracticable feature of uniformity the basis of legislation. It is on this rock that the American systems of registration have split. It ought to be clearly discerned by all who believe the true status of a nurse to be bound up indissolubly with the status of her training school.

